# Understanding HPV vaccination communication needs of parents of Turkish and Moroccan origin living in the Netherlands: a qualitative study

**DOI:** 10.1186/s12889-026-28910-z

**Published:** 2026-08-11

**Authors:** Enise Çayci, Thijs van den Broek, Anna P. Nieboer

**Affiliations:** Department of Socio-Medical Sciences, Erasmus School of Health Policy & Management, Erasmus University Rotterdam, Rotterdam, Netherlands

**Keywords:** HPV vaccination, Parental communication, Ethnic minorities, Semi-structured interviews, Culturally tailored interventions

## Abstract

**Background:**

HPV vaccination prevents HPV-related cancers, yet coverage in Europe remains low. In the Netherlands, uptake is particularly low among Turkish- and Moroccan-origin children, whose parents often have reservations about HPV vaccination, including mistrust, fear of side effects, and perceived information gaps. Understanding parents’ preferences and expectations regarding HPV vaccination communication is essential for developing tailored strategies that support informed decision-making about children’s vaccination.

**Methods:**

We conducted 30 semi-structured interviews with Turkish- and Moroccan-origin parents of children aged 7–9 years in the Netherlands between April 2024 and January 2025. Interviews were conducted in Dutch or Turkish, with informal Tamazight translation when needed.

**Results:**

Thematic analysis revealed a multidimensional discrepancy between official HPV vaccination information provision and parental expectations. Misalignment was perceived regarding communication setting, tone and framing of message, timing of information and messenger characteristics, which prompted some parents to rely on social networks and online sources. Parents suggested improvements, including timely, transparent and culturally sensitive communication delivered by trusted messengers familiar with their community.

**Conclusion:**

These findings underscore that effective HPV vaccination communication requires more than accurate information. Strategies must foster trust, engagement and comprehension, tailored to parental preferences. Tailoring content, formats, and sources to parental preferences may help reduce disparities in HPV vaccination uptake and support national and European immunization goals.

**Supplementary Information:**

The online version contains supplementary material available at https://doi.org/10.1186/s12889-026-28910-z.

## Background

Human papillomavirus (HPV) is the most common viral infection of the reproductive tract worldwide and a major cause of various cancers including oropharyngeal cancer and a range of anogenital cancers such as penile, anal, vaginal, vulvar and cervical cancers, affecting both men and women [1]. Vaccination is the most effective preventive measure against HPV-related cancers and precancerous lesions [2]. To prevent HPV infection, a one- or two-dose vaccine is recommended before the onset of sexual activity. The WHO prioritizes HPV vaccination for girls aged 9–14 years [3], while many national immunization programs (NIPs) have adopted gender-neutral vaccination strategies that include both girls and boys [4]. For example, in the Netherlands, HPV vaccination was introduced into the NIP for girls in 2009 [5], and extended to boys only in 2022 following an advice from the Dutch Health Council (Dutch: Gezondheidsraad) (Rijksvaccinatieprogramma) [6].

As part of Europe’s Beating Cancer Plan, the European Commission aims for 90% of girls to be vaccinated against HPV by 2030, along with significant increases in vaccination rates among boys [7]. Within the EU/EEA, HPV vaccine coverage rates averaged 62.2% in 2022, with only 9 out of 24 EU/EEA countries projected to reach the 90% target by 2030 [8]. The Netherlands is close to these averages: in 2025, the HPV vaccine coverage was 64.5% among girls and 60.9% among boys [9]. The fact that the Netherlands is not on track to meet the 2030 target underscores the need for more effective vaccination strategies. Importantly, significant disparities exist within the Dutch population. In the four largest Dutch cities (Amsterdam, The Hague, Utrecht, and Rotterdam), HPV vaccination uptake differs substantially by ethnic background, with uptake among girls of Turkish and Moroccan origin reported at 13% and 7%, respectively [10]. Today, these communities predominantly consist of Dutch-born descendants of earlier labor migrants representing two of the country’s largest communities with roots outside of Europe [11]. A 2025 report by the National Institute for Public Health and the Environment (RIVM)—the institute responsible for monitoring public health and providing guidance on vaccination policy and related public information campaigns in the Netherlands—alarmingly indicated that vaccination uptake among children of Turkish and Moroccan origin continues to decline [12]. Although there are also parents of Turkish and Moroccan origin in the Netherlands who view HPV vaccination favorably, many have strong reservations. These reservations are related to mistrust of health and governmental authorities, fears of long-term side effects, concerns about encouraging sexual activity, and the perception that children already receive too many vaccines [13].

Difficulties accessing and identifying reliable information about vaccination are also known to be a barrier to vaccination among ethnic minorities [14]. This has also been identified as a specific barrier to HPV vaccination, even among people of Turkish and Moroccan origin with a relatively open stance towards HPV vaccination [13, 15]. Communication and information processing are central to vaccination decision making [16]. In the Netherlands, parents are first informed of their child’s HPV vaccination eligibility through an invitation letter, automatically sent by the RIVM in the year the child turns 10. Vaccination itself is delivered through regional youth health services (Dutch: Jeugdgezondheidszorg) typically during group vaccination sessions organized locally. Beyond this invitation letter, RIVM campaigns provide general materials, such as brochures, websites, and other information on HPV vaccination and its side effects.

To better reach minority groups, RIVM has moreover implemented some tailored interventions, such as translating materials into various languages accessible through the National Immunization Program website, and, at the local level, some regional public health services (e.g., Rotterdam-Rijnmond) have implemented neighborhood-based approaches. These local initiatives actively engage parents through information sessions in the community to reduce barriers and increase vaccination uptake [17].

While such efforts represent important steps toward more tailored communication, the persistently low HPV vaccination rates among children with a Turkish or Moroccan background in the Netherlands raise questions about how well these strategies align with the needs and expectations of these children’s parents. Understanding these views is crucial for adapting future strategies to better meet their needs and ultimately enhance vaccine readiness.

Aligned with the WHO Immunization Agenda 2030’s principle of people-centeredness in immunization services [18], this study aims to explore how parents of Turkish and Moroccan origin in the Netherlands perceive current HPV vaccination communication and how they would prefer it to be delivered. Using a bottom-up qualitative approach, we identify parents’ preferred sources, formats, and content of information, and highlight how an improved alignment between HPV communication strategies and parental preferences could benefit trust, opportunities for dialogue, and, ultimately, HPV vaccination decisions.

## Methods

### Study design, participants and setting

We adopt an interpretivist qualitative approach to explore parents’ perceptions and expectations regarding HPV vaccination communication. The study was reported according to the Consolidated Criteria for Reporting Qualitative Research (COREQ) [19]. The completed checklist is provided in the supplementary materials.

Eligible participants were parents of children aged 7–9 years, of Turkish or Moroccan origin, living in the Netherlands. This age group was selected because children are invited for their first HPV vaccination around the age of 10. Note that some participating parents also had older children at the time of the study. A purposive criterion sampling strategy was used [20], in which parents were selected based on meeting the study’s inclusion criteria described above. Recruitment was conducted through multiple channels: flyers and direct outreach through the first author (EC)’s personal network, posts on social media, and direct outreach at community centers located in neighborhoods with a high density of Turkish and Moroccan-origin residents. At community centers, the first author approached parents directly to explain the study and assess interest. In some cases, parents directly expressed interest in participating. Flyers and social media posts included contact details, allowing interested parents to reach out directly to the research team. All interested parents were screened against the eligibility criteria before an interview was scheduled. Snowball sampling was additionally used, in which participants and personal contacts were asked to share information about the study with other eligible parents [21]. Study information was provided in Dutch or Turkish with informal translation for Moroccan-background participants who did not speak Dutch as their first language, if needed, before agreement to participation.

We recruited 31 participants, of whom 30 were included in the analysis. One interview was excluded: This respondent did not speak Dutch or Turkish, and no (Moroccan-)Arabic-, or Tamazight-speaking translator was available at the time of the interview. As the respondent indicated they could conduct the interview in Spanish, a language the first author also speaks, the interview was attempted in Spanish. However, communication in Spanish did not allow for sufficiently in-depth responses and the interview was excluded from analysis. Most participants were recruited from major Dutch cities with a smaller number residing in smaller municipalities, as the largest concentrations of Turkish- and Moroccan-origin communities reside in the major cities. The final sample included 19 parents of Turkish origin and 11 of Moroccan origin, including both mothers (67%) and fathers, primarily aged between 35 and 44 years. Participants had diverse educational and employment backgrounds. Most had completed higher education and their children varied in gender. An overview of the background characteristics of the participants is presented in Table 1.

**Table 1:** Demographic characteristics of study participants

Participant ID	Gender	Age category	Ethnic Origin	Child(ren)’s gender^a^	Education	Employment status	Sampling method	Language of interview
1	F	25–34	Turkish	F	Primary & Preparatory education	Not employed	Snowball	NL
2	F	35–44	Turkish	F	Higher education	Student	IN	NL
3	F	35–44	Turkish	F & M	Higher education	Employed	IN	NL
4	F	35–44	Turkish	F & M	Higher education	Employed	SM	NL
5	M	25–34	Turkish	F	Secondary & Vocational education	Employed	Snowball	NL
6	F	45–54	Turkish	M	Secondary & Vocational education	Employed	IN	NL
7	M	25–34	Turkish	F	Secondary & Vocational education	Not employed	Snowball	NL
8	F	35–44	Turkish	F	Higher education	Employed	IN	NL
9	M	35–44	Moroccan	F	Secondary & Vocational education	Employed	IN	NL
10	F	45–54	Turkish	F	Secondary & Vocational education	Employed	IN	NL/TR
11	F	35–44	Moroccan	F	Secondary & Vocational education	Employed	IN	NL
12	F	35–44	Turkish	F	Secondary & Vocational education	Employed	SM	NL
13	F	35–44	Turkish	F	Higher education	Employed	IN	TR
14	F	35–44	Moroccan	M	Higher education	Employed	CC	NL
15	F	45–54	Moroccan	F	Higher education	Not employed	CC	NL
16	F	25–34	Moroccan	F	Secondary & Vocational education	Not employed	CC	NL/BER
17	F	35–44	Moroccan	F	Primary & Preparatory education	Volunteer work	CC	NL/BER
18	F	25–34	Turkish	M	Secondary & Vocational education	Employed	IN	NL/TR
19	F	35–44	Turkish	F	Higher education	Employed	IN	NL
20	M	25–34	Turkish	F & M	Higher education	Employed	IN	NL
21	M	35–44	Turkish	F	Secondary & Vocational education	Employed	IN	NL
22	F	35–44	Moroccan	M	Secondary & Vocational education	Care provider	Street	NL
23	F	35–44	Moroccan	F	Higher education	Employed	IN	NL
25	M	35–44	Turkish	F	Secondary & Vocational education	Employed	IN	TR
26	F	35–44	Moroccan	F	Higher education	Employed	Snowball	NL
27	M	35–44	Moroccan	F	Secondary & Vocational education	Employed	Snowball	NL
28	M	35–44	Turkish	F	Higher education	Employed	Snowball	TR
29	M	35–44	Turkish	F	Higher education	Employed	Snowball	TR
30	F	25–34	Moroccan	F	Higher education	Employed	IN	NL
31	M	25–34	Turkish	M	Secondary & Vocational education	Employed	IN	NL

^a^Only includes children aged 7-9 as that is the inclusion criterion of the study. Notes: F = female; M = male; IN = informal network; SM = social media; CC = community centre; NL = Dutch, TR = Turkish, BER = Tamazight

### Data collection

The interview guide was developed from literature on vaccination decisions in ethnically diverse groups, while still allowing new themes to emerge during the conversations. Key thematic domains included: (1) Parental decision-making and factors influencing considerations for vaccinating their child [22–25]; (2) concerns and perceived risks about the HPV vaccine [26–28]; (3) information needs and perceived sufficiency, clarity, relevance of existing information [29–31] and, (4) preferred communication formats [24, 29, 32]. Semi-structured interviews were conducted face-to-face between April 2024 and January 2025 at a location chosen by each participant (e.g., home, community center) in Dutch or Turkish by the first author. For two participants who did not speak Dutch as their first language, an informal translator provided Tamazight translations as needed to ensure clarity.

All sessions were audio-recorded and lasted an average of 29 min (range: 16–72 min). Written informed consent was obtained from all participants before the interview. In addition, verbal consent was confirmed at the start of the audio-recorded interview. All participants received a 20-euro gift card for their time and contribution. The study was approved by the Erasmus School of Health Policy and Management Research Ethics Review Committee (ETH2324-0402).

### Data analysis

Audio recordings were transcribed verbatim using Amberscript and verified by the first author. Data were thematically analyzed following Braun and Clarke’s six-step process [33], chosen for its flexible yet systematic method of identifying and interpreting patterns of meaning. Analysis followed an inductive, data-driven approach. Although the interview guide was informed by existing literature, coding and theme development were derived from participants’ accounts rather than a pre-existing framework. Coding began at a semantic level, capturing participants’ explicit statements. This progressively moved towards a more latent level of interpretation during theme development, in line with the study’s interpretivist paradigm to capture the underlying meanings and assumptions shaping parents’ perspectives. Coding was initially organized around the thematic domains underlying the interview guide. Transcripts were then reviewed in full and additional quotes were generated inductively for content considered relevant to the study beyond these initial domains. Steps included: (1) data familiarization, (2) generating initial codes, (3) searching for themes (4) reviewing themes, (5) defining and naming themes, and (6) producing the final report. Initial coding was conducted by the first author, with the co-authors (AN, TB) contributing to theme development, review, and interpretation. Consensus on theme development was reached through iterative discussions with the research team. Data extracts played an active role throughout theme development. During analysis, specific extracts were used by the research team to discuss codes and candidate themes and to assess whether they were supported across participants. Additional extracts were selected after themes had been finalized to illustrate the final themes. Analyses were conducted using Atlas.ti software (version 25.0.1) until thematic saturation, defined as the point at which no new themes emerged from the interviews and confirmed through ongoing team discussions.

## Results

**Fig. 1:**
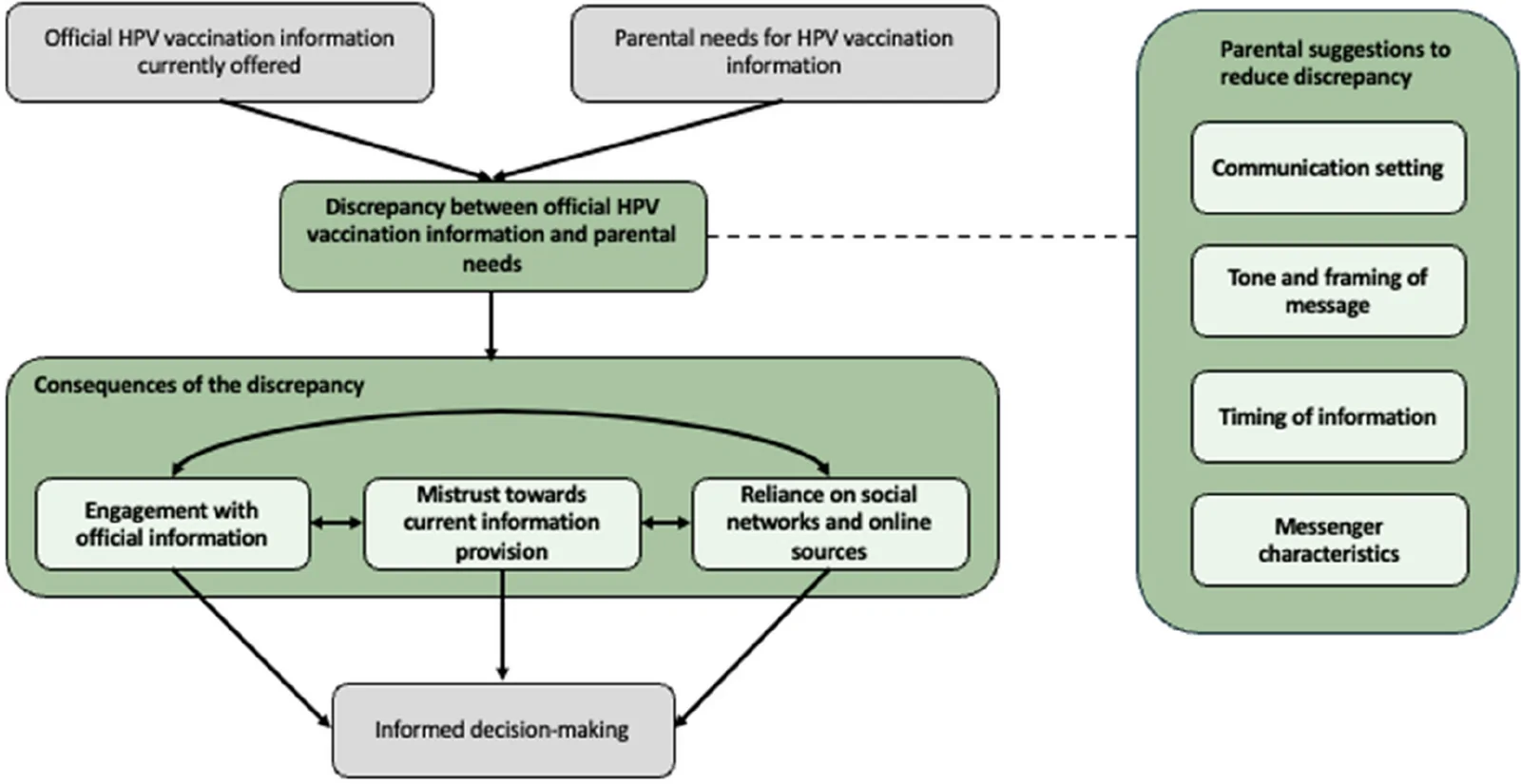
Conceptual framework derived from the thematic analysis illustrating the relationships between contextual elements and outcome of the thematic process (grey), overarching themes (dark green) and subthemes (light green). The figure shows how discrepancies between official HPV vaccination information and parental information needs influence engagement with official information, trust in current information provision and reliance on social networks and online sources, ultimately shaping informed parental decision-making. It also illustrates communication strategies identified to reduce these discrepancies

Thematic analysis identified three main themes: (1) discrepancy between official HPV vaccination information and parental needs; (2) consequences of this discrepancy, including three subthemes: engagement with official information, mistrust towards current information provision, and reliance on social networks and online sources; and (3) parental suggestions to reduce discrepancy including four subthemes: communication setting, tone and framing of message, timing of information and messenger characteristics. These themes and subthemes are presented below and summarized in Fig. 1.

### Discrepancy between official HPV vaccination information and parental needs

Parents emphasized that HPV vaccination information should be interactive and allow dialogue, rather than being delivered passively through letters or flyers. The impersonal, formal nature of current communication, along with repeated reminders, often felt misaligned with these expectations. Several parents reported that messaging seemed coercive or fear-driven, provoking distrust and even aversion toward HPV vaccination. As one Turkish-Dutch mother explained:

“People are being forced. […] Panic is spread first, and then ignorance is taken advantage of.” – Participant 3

This sense of coercion reflects a broader pattern in which fear-based messaging, rather than fostering informed choice, provoked resistance and regret among parents who felt pressured rather than informed.

Timing and framing further contributed to the discrepancy. Parents’ usual form of communication regarding HPV vaccination is a letter sent by the RIVM. Many parents described having received “*only a brief flyer or letter*”, or nothing at all if their children were not yet eligible, which they perceived as insufficient to make an informed decision. Parents considering vaccination for sons experienced additional uncertainty, because official messaging was historically centered on girls. This made the recent recommendation for boys seem inconsistent with prior communication. One Moroccan-Dutch mother who had already decided to vaccinate her daughter, described specific hesitance regarding her son:

*… the only thing we’re hesitant about is that it’s only been recommended for boys for two years now. There hasn’t been much research done on boys, so I think… is my child kind of a test subject? – Participant 30.*

Limited understanding of HPV further contributed to hesitation. Parents emphasized the need for comprehensive information for both genders. As one Turkish-Dutch father describes:

*"Cervical cancer… You know, a boy doesn't have a uterus. So, then you start thinking, why should I vaccinate my child against that? So yes, I don't really know. […] I think the information just needs to be complete for both [boys and girls]." – Participant 7.*

This illustrates a specific gendered gap in HPV vaccination because official messaging has historically centered on cervical cancer prevention. Parents of sons often lacked a comparable rationale for vaccinating boys, leaving the relevance of vaccination for non-uterus-related HPV risks underexplained.

Messenger credibility also shaped engagement, reflecting another dimension of the discrepancy. While some trusted the preventive health centers that their children visit frequently for check-ups, growth monitoring, and vaccinations until adulthood, others viewed these institutions as overly closely tied to government authorities. For these parents, this formal association reduced credibility, making centers less suitable messengers of information, as it created a gap between parents’ expectations for trustworthy, independent messengers and the characteristics of current messengers. As one Moroccan-Dutch mother explained:

*“The Center for Youth and Family [municipal center for preventive child health and parenting support], yes, they’re like friends to me, so to speak. But there is also a very large group that doesn’t experience it that way and doesn’t see it like that. So, the information coming from there would not be received wholeheartedly.” – Participant 30.*

Altogether, these findings highlight discrepancies regarding setting, tone, timing, clarity, gender framing and messenger credibility. This misalignment shapes engagement, trust, and confidence in official information.

### Consequences of the discrepancy

The discrepancy influenced parents’ willingness to engage with HPV vaccination communication and raised concerns about the trustworthiness and credibility of sources, leading many parents to rely more on social and online networks for guidance when making vaccination decisions: 

#### Engagement with official information

Despite the perceived deficiencies, most parents expressed a need for official HPV vaccination information to make informed decisions, indicating a willingness to engage with communication strategies. However, the discrepancy influenced the extent of engagement. Particularly parents who were firmly opposed to vaccination avoided official materials altogether. For these parents, receiving the information offered was seen as unnecessary or even unwelcome. As one Turkish-Dutch mother explains: *“I don’t pay attention to it. If I see it, I just skip it. I’m not even invited anymore for the children to get vaccinated, because I’ve made that very clear. So no, I really don’t engage with it.” – Participant 3.*

This tension highlights that providing official information does not automatically lead to engagement. Instead, parents’ perceptions of its relevance, completeness and credibility shape whether and how they interact with it. Consequently, the discrepancy not only affects trust in official sources and information provision but also drives parents toward alternative sources to fill perceived gaps in information. 

#### Mistrust towards current information provision

Parents’ mistrust towards official HPV vaccination communication stems from the discrepancy between their expectations and the way information is currently communicated. A key dimension of this discrepancy is messenger credibility. Some parents expect information to come from independent and trustworthy sources, but real and perceived ties between official messengers and government or industry can undermine this expectation. These parents expressed doubts about the trustworthiness of authorities and the intentions behind the information they provide. One Moroccan-Dutch mother explains:

*“I have little trust in the government anyway, and no, I think most of it is just about money. The health of citizens isn’t really a priority for either the government or the manufacturers.” - Participant 14*

This mistrust extended to the completeness and transparency of information, including whether reported side effects were the only ones that could occur. As one Turkish-Dutch mother puts it: *“I would want to look into it myself, because I have this feeling, maybe a bit distrustful, but the moment I receive that information directly, it feels one-sided to me.” – Participant 12.*

These perceptions highlight a mismatch between parents’ need for clear, comprehensive information and the current information provision that they perceive as selective and fear-driven. Overall, mistrust of official communication is not a general sentiment, but a direct consequence of the misalignment between parental expectations for credible, transparent messengers and the current HPV vaccination communication.

#### Reliance on social networks and online sources:

In response to the perceived lack of trustworthiness of official information and its misalignment with parental preferences, many parents turned to social networks and online sources, which they perceived as more trustworthy and relevant. Several participants emphasized that they actively sought information beyond official channels, reflecting a broader mistrust of government-provided information. One Turkish-Dutch mother explained:

“I would also ask around in my social circle, people with children and those who have experience, or people who administered the vaccine and have heard about side effects from family members. I would also do my own little research. I wouldn't rely solely on information from the government, of course.” – Participant 6.

Online sources were used to complement official information, but the abundance of content sometimes complicated decision-making. Some parents described becoming confused or overwhelmed, which could ultimately delay or prevent vaccination decisions:

“You don’t get much information about it. You do find some things on the internet. I read that, of course, but for me, it doesn’t really make it easier to make the right decision." – Participant 29.

Even before receiving official information about HPV vaccination, some parents anticipated relying on social networks and online sources. This highlights that when official communication is perceived as insufficiently trustworthy, incomplete, or delayed, families may turn to alternative sources, increasing their risk of exposure to misinformation that could negatively influence HPV vaccination decision-making.

### Parental suggestions to reduce the discrepancy

Parents suggested adjustments in *communication setting*, *tone and framing of message, timing of information and messenger characteristics* to reduce the discrepancy between official HPV vaccination information and their preferences. 

#### Communication setting

Parents emphasized that HPV vaccination information should be interactive, allowing for dialogue, rather than passive one-way communication. A recurring suggestion was to organize information sessions for parents, where they could discuss questions in person with experts with appropriate characteristics (described in more detail under *Messenger Characteristics*). One Moroccan-Dutch father explained how this could provide reassurance prior to the vaccination decision:

"For example, the information, before even offering the vaccine, you could give parents the choice and invite them to an information evening. I would find that interesting, because then you can also ask critical questions." – Participant 9.

Beyond expert input, parents highlighted the importance of peer contact during these sessions, underscoring that vaccination decisions are embedded within social networks as much as in professional advice.

Preferences for settings varied. Schools were mentioned by some as convenient. Other parents emphasized the value of face-to-face interactions in preventive health centers which allow for spontaneous dialogue and direct access to professionals to ask questions. However, some parents believed the ties of these centers to governmental authorities would undermine perceived independence and trustworthiness. These parents, therefore, preferred community-based, informal gatherings facilitated by familiar figures, such as local community members which were perceived to foster trust, openness and engagement. One Turkish-Dutch mother explained:

“I think it should be a kind of informal, relaxed coffee moment-like gathering in the neighborhood, community, or association, something like that, where you basically have a key figure in the area, someone who is already volunteering there. For example, a Moroccan woman who then organizes an information session together with the institution… I think key figures are important, because immigrant groups usually come to neighborhood associations and end up going to that specific person.” – Participant 19.

Notably, the specific example of “a Moroccan women” as a trusted key figure foreshadows a theme discussed in more depth below (see Messenger Characteristics). For these parents, communication setting and messenger identity are closely intertwined, as trust in a setting often depends on who represents it. 

#### Tone and framing of message

 To foster trust and ensure clarity, messages should be clear, accessible and balanced, avoiding overly technical language. This would help parents understand and engage with the content. One Moroccan-Dutch parent suggested:

"You can describe it in a very complicated and beautiful way, but I actually think the simpler and more 'Jip and Janneke language’ [Dutch expression referring to very simple, child-friendly language, based on a popular Dutch children’s book series] you use, the better it will reach the average citizen…" – Participant 30.

Parents also emphasized how they preferred messaging to be balanced when directed at children. They argued that vaccination information should neither oversimplify the benefits nor create what they perceived as false security. Although the vaccine offers substantial protection, HPV-related risks are not fully eliminated, and some parents felt that both children and parents should be made aware of this. One Turkish-Dutch mother explained that messages should clearly balance the protective effects with the remaining risks, making explicit that vaccination does not guarantee full protection against HPV or other STDs:

“Imagine, education [schools] provides information like: if you get the shot, you’re protected. But maybe it should be explained differently: if you don’t get the shot, then you have a higher chance or risk. […].I think schools should clearly explain it’s not that you’re vaccinated and therefore completely safe. No, you’re vaccinated, but there are still significant risks, so think carefully about what you do.” – Participant 6.

#### Timing of information

 Many parents reported that they typically received HPV vaccination communication only when their child became eligible, leaving little time to reflect or ask questions. For parents of children not yet eligible, HPV vaccination was often seen as “*side issue*” with low salience and of which they had only limited knowledge. The timing of information was therefore seen as a key factor contributing to the perceived discrepancy, as late provision undermined parents’ understanding and affected their decision-making once the vaccination invitation arrived. Consequently, parents suggested that information about the HPV vaccine could be provided earlier in childhood. As one Moroccan-Dutch father noted:

“I do believe there are vaccines that are good for your children. But then I think where’s the proper information beforehand? Before your child even receives the shot, someone should sit down with you and say: this is what we’re going to do, this is why, and make sure it’s someone who actually knows what the vaccine is for. That’s often missing.” – Participant 9.

#### Messenger characteristics

Parents reported that current HPV vaccination messengers often do not meet their expectations, contributing to mistrust and uncertainty. This reflects the broader discrepancy in messenger credibility between parental expectations for credible, transparent messengers and the official communication they currently receive. To address this, parents identified key characteristics of what they saw as trustworthy communicators. They should be honest, transparent, knowledgeable and independent, able to provide in-depth, clear explanations, address questions beyond scripted messages, engage with critical concerns in a reassuring manner, and be independent and objective. One Turkish-Dutch father explained:

“Someone who has knowledge of it and someone who understands it. Not someone who just quickly reads a book or something. That’s not how it works. No, really someone who goes deeper into it”.—Participant 31.

Cultural and linguistic familiarity was also valued. Parents found messengers who understood their community context more credible, trustworthy and accessible. One Turkish-Dutch mother described:

“Because a Turkish speaker would inspire more trust than a Dutch speaker or doctor. And if that Turkish speaker is also a doctor or works in the medical field, then they would trust it more easily”.—Participant 19.

Additionally, independence from governmental or industry influence was emphasized. Again, perceived ties to authorities or pharmaceutical companies were seen as undermining neutrality, reinforcing the discrepancy between parental expectations and current official communication.

One Turkish-Dutch mother explained:

“It must be an organization that is independent from the government and also trustworthy in society. …Not just some companies you've never heard of that conduct a study and say, ‘We did research, here, it’s good.’ It needs to be a long-established company to come across as somewhat trustworthy”. -Participant 8.

Parents’ suggestions illustrate that messenger credibility is central to reducing the perceived discrepancy, supporting trust, engagement and informed decision-making. Messengers who combine professional expertise, transparency, independence, and cultural/linguistic familiarity are essential to effectively deliver HPV vaccination to these parents.

## Discussion

While international studies have explored the information needs of hesitant, ethnically diverse parents (e.g., [29]), and Dutch research has highlighted the importance of culturally tailored communication for groups like people of Turkish and Moroccan origin (e.g., [34]), little is known about how these parents experience current HPV vaccination information, despite them being the primary decision-makers for their children’s HPV vaccination. This study addresses this gap by exploring parents’ experiences with HPV vaccination communication and their preferences, to inform strategies supporting informed decision-making. Our findings show that parents experience a clear discrepancy between official HPV vaccination information and how they would prefer it to be delivered. This discrepancy influenced parents’ willingness to engage with HPV vaccination communication and raised concerns about the trustworthiness and credibility of sources, leading many parents to rely more on social and online networks for guidance when making vaccination decisions. In response to these challenges, parents offered suggestions for reducing the discrepancy, including preferences regarding communication settings, tone and timing of messages, and characteristics they expect messengers to have. Together, these findings highlight potential strategies for improving HPV vaccination communication to better align with parents’ needs and preferences. The connections between these themes are summarized in Fig. 1.

### The perceived discrepancy

The findings reveal a multidimensional discrepancy between current official HPV vaccination communication and parents’ preferences. Mismatches in communication setting, tone and framing, timing and messenger characteristics reduce engagement and lead parents to disengage from current initiatives. Despite these shortcomings, parents still expressed a need for HPV vaccination information, indicating willingness to engage with communication better aligned to their preferences. As a result of the discrepancy, parents often develop mistrust toward the provided information and seek alternative sources, such as social networks and online platforms, which are not always reliable. Exposure to informal sources is known to affect vaccination intention [35–37], thereby undermining informed decision-making. Overall, our findings align with broader evidence in health communication, showing that inequalities in access, exposure, and use of health communication, can influence disease knowledge, health beliefs, and preventive behaviors [38, 39]. Furthermore, delayed or poorly targeted communication can undermine vaccine confidence [40].

### Implications for practice and policy

#### Communication setting

Parents emphasized interactive communication, allowing dialogue rather than one-way delivery via letters or flyers. Providing information at preventive health centers, perceived as practical sites for face-to-face conversations, reflects the importance of interactions with health professionals in addressing parents’ questions and concerns [41, 42]. However, some parents perceive such settings as undermining trust and engagement due to their ties with the government. Community-based sessions led by familiar figures may offer a complementary approach. Prior research showed that participatory, culturally tailored approaches strengthen trust and vaccine confidence by aligning messaging with local norms, values, and trusted voices, underscoring the importance of multi-setting, context-sensitive strategies to address diverse parental preferences [43]. Face-to-face educational interventions help parents understand vaccine importance, access services, address hesitancy or concerns about vaccine safety or efficacy [44]. Our findings suggest that combining center-based accessibility with culturally familiar community-based settings to foster trust may better accommodate heterogeneity in parental preferences and trust-related dynamics. Across settings, sessions should allow two-way dialogue and peer exchange to support informed decision-making.

#### Tone and framing of message

Parents preferred clear, balanced and accessible communication, avoiding overly technical language that can create confusion or disengagement. Experts have highlighted that messages heavy in scientific jargon can exceed recipients’ cognitive capacity, leading to reliance on cues such as source credibility without fully understanding the information [45]. When parents who are already hesitant perceive vaccination messages as overly technical, this may exacerbate uncertainty or reduce engagement with the information. Communication that uses plain, accessible language, presents both benefits and risks in a balanced manner and avoids one-sided framing may reduce this cognitive burden. This could allow parents to process information on its merits rather than relying solely on peripheral cues such as source credibility. This, in turn, can support genuine comprehension rather than superficial compliance and foster trust grounded in transparency rather than assumed credibility. 

#### Timing of information

The results revealed that late provision of HPV vaccination information did not align with parents’ preferences. Receiving information only at eligibility, or not at all beforehand, limited their ability to reflect, ask questions or make informed decisions. Timely, interactive communication could reduce uncertainty and knowledge gaps, which are closely linked to susceptibility to misinformation [46]. This underscores the importance of timely dissemination of vaccine information as strategy to reduce the perceived discrepancy between parental needs and current official communication. 

#### Messenger characteristics

Credibility, transparency, independence and cultural or linguistic familiarity are essential for trusted communication. These characteristics align with well-established determinants of trustworthiness: ability (competence and expertise), benevolence (caring, goodwill and commitment), and integrity (honesty, transparency and fairness) [47, 48]. While these traits support trust, our findings indicate that shared cultural or linguistic backgrounds further enhance trust among hesitant parents of these origins. This nuance highlights that trust depends not only on perceived ability, benevolence or integrity, but also on relational and contextual alignment between messenger and recipient. Given the continued decline in vaccination uptake in this group, prioritizing trust-building initiatives is particularly urgent [12].

Our findings also point to a gender-specific dimension of the discrepancy that warrants further attention. Rather than focusing solely on cervical cancer as done in the past, current campaigns in the Netherlands emphasize the range of HPV-related cancers affecting both boys and girls. Our findings, however, suggest some parents’ perceptions have not yet caught up with this shift. This pattern is not unique to Dutch context, nor to Turkish and Moroccan origin parents specifically. Similar gaps have been reported elsewhere, for example in the UK. There, awareness and uptake among boys remain limited, shaped by the historical framing of HPV vaccination as a “girls” vaccine, persistent gendered misconceptions and limited public awareness of boys’ eligibility [49]. In Dutch context, the gap between updated official framing and parents’ perspectives on the matter may be partially explained by the fact that the vaccination strategy focused on both boys and girls was just introduced in 2022. Consequently, there may be a possible lag in how quickly updated messaging reaches and resonates with parents. For parents of Turkish and Moroccan origin specifically, this lag may be further compounded by the broader discrepancy identified throughout this study, between official information and their communication preferences. This could slow how quickly updated messaging reaches and is absorbed by these communities. Addressing this gap requires communication that explicitly correct the misconception that HPV is a female-specific concern, reinforcing that boys are also vulnerable to HPV-related disease.

Parents expressed diverse preferences regarding HPV vaccination communication, reflecting heterogeneity within Turkish- and Moroccan-Dutch communities. Many concerns and needs identified align with findings from other populations (e.g., [29]), indicating room for improvement in communication for both the general public and specific groups. Broad “catch-all” non-tailored communication strategies may be less effective risk exacerbating vaccination inequities (cf. [32]). The mechanisms identified in this study include the role of source credibility, the cognitive burden associated with technical communication and reliance on informal networks in the context of institutional mistrust. These mechanisms may also be relevant for other ethnic minority communities facing similar communication challenges, both within the Netherlands and internationally. Therefore, we recommend a bottom-up approach that directly involves parents in the development of communication strategies, with our findings providing an important first step toward parent-centered HPV vaccination communication.

### Strengths and limitations

This study provides in-depth bottom-up qualitative insights of Turkish- and Moroccan-origin parents’ perceptions, needs and preferences regarding Dutch HPV vaccination communication. Recruiting mothers and fathers with diverse educational backgrounds and (HPV) vaccine attitudes ensured rich qualitative materials reflecting heterogeneity within and between these communities.

The study has limitations. The sample, while purposive and diverse, may not fully represent all parents with Turkish and Moroccan origin in the Netherlands, particularly, those of practically oriented education backgrounds. Parents with different educational backgrounds may vary in how they access, evaluate or articulate concerns about official vaccination communication thus, our sample may not fully capture this diversity. Additionally, most participants were recruited from major Dutch cities, with only a small number residing in smaller municipalities. As a result, the specific communication preferences and concerns identified in this study may be less representative of parents in smaller municipalities. While the first author’s shared linguistic and cultural background may have fostered trust, social desirability bias may have affected participants’ responses, particularly on sensitive topics such as sexuality and vaccination decisions. This may have led some parents to downplay reservations or present more favorable views of official communication than they privately hold. Interviews were conducted in Dutch and Turkish with informal translation (Tamazight) provided for two participants who did not speak Dutch as their first language. While this approach allowed for broader participation, reliance on informal translators may have introduced interpretive variation or filtering of participants’ responses, as informal translators may have unintentionally simplified, summarized or softened sensitive content.

## Conclusion

This study demonstrates that parents of Turkish and Moroccan origin in the Netherlands perceive a clear multidimensional discrepancy between current HPV vaccination communication and their preferences for information delivery. Key dimensions include communication setting, tone and framing of messages, timing of information, and messenger characteristics. Parents value interactive, culturally familiar, and transparent approaches that allow dialogue and peer exchange. They prefer messages to be timely, balanced and clear. Failure to meet these preferences contributes to mistrust and reliance on social and online sources, which may expose parents to misinformation, reinforcing the mechanism of mistrust. While some preferences highlighted in the current study overlapped with previously reported findings for other demographic groups, the heterogeneity within communities in this study underscores the need for tailored, culturally sensitive strategies. Our findings underscore the importance of a bottom-up approach that actively involves parents in designing communication, ensuring comprehension and trust, supporting informed decision-making, strengthening vaccine confidence, and ultimately, contributing to national and European immunization targets.

## Supplementary Information

Supplementary Material 1.

Supplementary Material 2.

Supplementary Material 3.

## Data Availability

The qualitative data used in this study are not publicly available, as they form part of an ongoing project entitled "Applying a Gender Perspective to Ethnic Differences in HPV-Vaccination Uptake in the Netherlands (GETVACCINL)," and to safeguard the privacy of participants. In accordance with the terms of participant consent and ethics approval, these data will not be made available for reuse.

## Abbreviations

CBS: Statistics Netherlands
COVID-19: Corona Virus Disease 2019
GGD: Municipal Public Health Services (Gemeentelijke Gezondheidsdiensten)
HPV: Human Papilloma Virus
NIP: National Immunization Program
RIVM: National Institute for Public Health and the Environment (Rijksinstituut voor Volksgezondheid en Milieu)
WHO: World Health Organization
