# Supplementary material for: Understanding HPV vaccination communication needs of parents of Turkish and Moroccan origin living in the Netherlands: a qualitative study
Source: BMC Public Health. 2026 Aug 11;26:3256. doi: 10.1186/s12889-026-28910-z (PMC13746602; doi:10.1186/s12889-026-28910-z)
Supplement: Supplementary file 3 [file 12889_2026_28910_MOESM3_ESM.docx]

**Supplementary material 1:** Interview guide in English

| Topic | Prompts |
| --- | --- |
| Introduction | - Conversation |
| Perspectives on HPV vaccination | |
| Parental decision-making  Influencing actors | *Are you considering having your child(ren) vaccinated against HPV?*   - What factors play a role in this decision? - Why do these factors influence your decision? |
| Perceived risks and concerns  Vaccine safety perception | *Do you have concerns about the HPV vaccination for your child(ren)?*   - What are you concerned about? - Why are you concerned about this? |
| Gender differences in vaccination decisions  (Cultural) norms | *Would you make a different decision for your son than for your daughter?*   - Could you explain why you think this?   Thorough Elaboration  If participant mentions difference:   - What do you think this difference is related to? |
| Perceptions of current HPV vaccination communication | |
| Information needs and perceived sufficiency  Accessibility of information  Clarity and relevance of current information  Sources of information  Trustworthiness  Preffered communication formats | *Do you feel like you have sufficient information about the HPV vaccination to make a decision for your child(ren)?*   - What type of information about the vaccine would you like to receive?   - Why would this work for you? - In what way should this information be communicated to you?   - Why? - From whom would you like to receive this information?   - Why?     - Trust: Whose information would you trust then?   If participant indicates that they already have sufficient information:   - What information did you obtain? - Through which channels did you receive it? - From whom did you receive this information? - What made you trust the information? |
| Gender-sensitive communication  Targeted health messaging | *Do you think communication about HPV-vaccinarion should be targeted differently towards parents of boys versus parents of girls?*     - Why do you think this [based on their answer]?   - Dive deeper into the motivations if needed. |
| Closing | *We have arrived to the end of the conversation*   - Is there anything you would like to add? - Have we discussed all important aspects of this topic? - Are you satisfied with the interview? - Thank you for sharing your opinion?   *End audio-recording* |

**Supplementary material 2:** COREQ checklist
